# Association of premarital medical check-ups and folic acid knowledge, attitudes, and practices with the occurrence of neural tube defects in Guangxi, China (2009–2023)

**DOI:** 10.7189/jogh.16.04129

**Published:** 2026-06-26

**Authors:** Xianghong Liu, Yang Peng, Jing Zhu

**Affiliations:** 1 Guangxi Clinical Medical Research Center for Birth Defects, Guangxi Key Laboratory of Reproductive Health and Birth Defects Prevention and control, Maternal and Child Health Hospital of Guangxi Zhuang Autonomous Region, Nanning, China; 2 College of Acupuncture-Moxibustion and Tuina, Guangxi University of Chinese Medicine, Nanning, China

## Abstract

**Background:**

Neural tube defects (NTDs) are severe congenital malformations that cause significant foetal mortality and lifelong disability. Preconception folic acid supplementation has been proven effective in reducing the risk of NTDs. In 2009, China launched a national folic acid supplementation programme, which was further strengthened in Guangxi, China, by the implementation of a free premarital medical check-up policy in 2010. In this study, we analysed NTDs incidence trends in Guangxi (2009–2023) to evaluate the impact of these integrated interventions.

**Methods:**

We conducted a descriptive epidemiological study utilising surveillance data from Guangxi on premarital medical check-up (PMC) rate, folic acid supplementation rate, compliance rate, awareness rate, and the number of NTDs cases from 2009 to 2023. We used the Cochran–Armitage trend test to assess temporal trends, and Spearman's rank correlation and linear regression analyses to examine associations between PMC rate, folic acid-related indicators, and the NTDs incidence rate.

**Results:**

We observed significant increasing trends in the PMC rate, folic acid supplementation rate, compliance rate, and awareness rate (*P* < 0.001, respectively) from 2009 to 2023. Conversely, the NTDs incidence rate demonstrated a significant decreasing trend (χ^2^ = 365.556, *P* < 0.001). Spearman correlation analysis showed significant negative correlations between the NTDs incidence rate and the PMC rate (r = −0.986, *P* < 0.001), folic acid supplementation rate (r = −0.932, *P* < 0.001), and folic acid awareness rate (r = −0.976, *P* < 0.001). Linear regression models indicated that increases in the PMC rate (*β* = −0.063, *P* < 0.001) and the folic acid supplementation rate (*β* = −0.090, *P* < 0.001) were significant predictors of a decrease in the NTDs incidence rate.

**Conclusions:**

The findings highlight the pivotal role of premarital medical check-ups and folic acid supplementation in preventing NTDs. The sustained promotion and reinforcement of these public health interventions are essential for further reducing the burden of birth defects and improving population health outcomes. The ‘Guangxi model’ of integrated premarital and reproductive health services offers valuable insights in this context.

Neural tube defects (NTDs) are severe congenital malformations resulting from the failure of neural tube closure during early embryogenesis, primarily comprising anencephaly, spina bifida, and encephalocele [1,2]. These defects represent a significant global public health challenge and are associated with high rates of miscarriage, stillbirth, neonatal death, and long-term disability among survivors, thereby imposing substantial burdens on healthcare systems, families, and societies [3,4].

Regional birth defect surveillance data shows that the incidence of NTDs among perinatal infants in Guangxi, China, declined markedly from 2009 to 2022 [5]. Compared with other regions in China, Guangxi, located in its south, has historically exhibited lower NTD incidence levels than the traditional high-prevalence areas in northern China; however, at the national level, its incidence remains higher than the low-endemic levels observed in some economically developed regions [6,7]. These findings indicate substantial regional disparities in the NTD disease burden and progress in prevention and control across China [8].

From a global perspective, NTD incidence varies widely across countries and regions. According to evaluations across different World Health Organization (WHO) regions, the overall incidence of NTDs ranges from approximately 0.3 to 199.4 per 10 000 births. NTDs continue to occur at very high rates in certain low-income settings or regions with incomplete surveillance systems, whereas countries and regions that have implemented effective folic acid supplementation strategies and mandatory food fortification policies report substantially lower incidence levels [9,10].

Premarital medical check-ups (PMC) are an integral component of preconception care, as they offer premarital health assessments, counselling on reproductive health, and screening for conditions potentially affecting marriage and offspring health [11–13]. Concurrently, periconceptional folic acid supplementation is a well-established evidence-based intervention for reducing the risk of NTDs [14]. Folic acid plays a critical role in DNA synthesis, methylation processes, and cellular development; its deficiency can disrupt these essential metabolic functions, contributing to the pathogenesis of NTDs [15].

In 2009, the Chinese government launched the national Folic Acid Supplementation for Prevention of NTDs project, providing free folic acid supplements to women of reproductive age. Guangxi Zhuang Autonomous Region implemented this initiative and further introduced a comprehensive free PMC policy in February 2010. The Guangxi PMC programme, therefore, represents a comprehensive public health strategy aimed at building a robust premarital and preconception health platform. Its primary goal extends beyond targeting any single disease, focusing instead on the integrated prevention and control of birth defects and the improvement of birth population quality through a ‘one-stop’ service model.

Beyond serving as a regional implementation example of the PMC programme, Guangxi Zhuang Autonomous Region was selected as the study area for its important historical and sociodemographic relevance. It also has a relatively high proportion of ethnic minority populations and is characterised by substantial heterogeneity exists in terms of economic development, educational attainment, and allocation of healthcare resources. Previous studies have shown that such sociodemographic and economic disparities can substantially influence maternal utilisation of periconceptional healthcare services, as well as adherence to preventive measures such as folic acid supplementation [16,17]. Specifically, some rural and socioeconomically disadvantaged areas in Guangxi have relatively lower rates of systematic maternal management, limited accessibility to health education, and weaker primary healthcare capacity. Long-standing inequalities in maternal and child health service utilisation between urban and rural areas and across different prefectures have also been documented [18]. This context provides a realistic setting for evaluating the effectiveness of institutionalised public health interventions under complex sociodemographic conditions.

Here, we analysed trends in PMC rates, folic acid knowledge, attitudes, and practices (KAP) indicators, and NTDs incidence in Guangxi from 2009 to 2023. We examined the associations between these factors and evaluated the combined impact of PMC and folic acid promotion on reducing NTD rates. We aimed to inform the refinement of public health strategies for birth defect prevention.

## METHODS

### Data sources

We obtained data from the Guangxi Maternal and Child Health (MCH) surveillance system and annual MCH statistical reports for 2009–2023. We collected the number of marriage registrations, PMC participants, total perinatal births, NTD cases, and data on folic acid supplementation (*i.e.* the number of women who should take folic acid, those who took it, those compliant with the regimen, and those aware of its benefits). We derived birth defect data from a long-term routine surveillance system.

While such administrative surveillance data offer advantages in population coverage and temporal continuity, they are also subject to potential biases, including underreporting and misclassification. To minimise these biases, we ensured that birth defects surveillance in Guangxi followed standardised national guidelines. We based case diagnoses on prenatal screening, prenatal diagnosis, and postnatal clinical or imaging examinations, followed by multilevel review and quality control procedures. Furthermore, we maintained relatively stable surveillance protocols and diagnostic criteria during the study period, which helped reduce systematic bias due to changes in monitoring practices. 

### Intervention and quality control

We evaluated two integrated public health interventions – the PMC programme and the periconceptional folic acid supplementation protocol.

#### Guangxi PMC programme

The Guangxi PMC programme provided a unified, free health service, in which participants receive a single, comprehensive check-up at integrated marriage and childbirth service centres. The service protocol was personalised based on marital and pregnancy status. For unmarried, non-pregnant couples (as primary targets), an integrated package of premarital and preconception health checks was provided. For women components included health education, history taking, physical examination, vaginal secretion tests, comprehensive blood tests (complete blood count, liver/kidney/thyroid function), infectious disease screening, TORCH antibody testing, and gynaecological ultrasound. For men components included health education, history taking, physical examination, complete blood count, haemoglobin electrophoresis, liver/kidney function tests, and infectious disease screening. During post-check-up services, all participants received risk assessment, counselling, and health guidance. Folic acid was distributed on-site. Individuals with positive screening results (*e.g.* for thalassemia, HIV/syphilis) were referred through a dedicated tracking system.

#### Folic acid supplementation protocol

The folic acid supplementation was implemented in accordance with the national Folic Acid Supplementation for Prevention of Neural Tube Defects programme [19]. The target population were women planning pregnancy, with supplementation recommended for three months before conception and during the first trimester. The dosage was standardised as follows: a standard dose (0.4 mg once daily) was prescribed for average women planning pregnancy, while a a high dose (4 mg once daily) was prescribed for high-risk women planning pregnancy (defined as those with a history of a previous NTD-affected pregnancy or those taking anti-epileptic drugs). The programme management ensured a sufficient supply for the entire recommended period.

### Data quality assurance

The data were generated under a province-level public health programme with a rigorous quality control system. All information regarding folic acid distribution and follow-up was required to be entered into the MCH Information Management System within 48 hours. A three-tiered (county, city, province) supervision and evaluation mechanism was implemented, involving quarterly county-level audits, semi-annual city-level guidance, and unannounced provincial inspections. This system combined real-time digital monitoring with on-site verification to enable continuous oversight and feedback concerning data completeness and accuracy, with prompt correction of identified errors. Furthermore, a mandated monthly follow-up was conducted by healthcare providers to encourage adherence and record compliance, which maximised data quality and helped mitigate surveillance bias.

### Case definition and indicators

We identified NTD cases (anencephaly, spina bifida, encephalocele) and classified them according to the standards outlined in the Chinese MCH surveillance manual [20]. To avoid double-counting and to reflect clinical severity and public health concerns, we adopted the hierarchical classification principle, categorising cases with multiple defects as anencephaly > spina bifida > encephalocele [21].

We calculated the key indicators as follows, where perinatal infants refer to live births, stillbirths, and foetal deaths from 28 weeks of gestation to within 7 days after birth:

- PMC rate (%) = (number of PMC participants / number of marriage registrations) × 100;
- Folic acid supplementation rate (%) = (number of women taking folic acid / number of women who should take folic acid) × 100;
- Folic acid compliance rate (%) = (number of women compliant with folic acid regimen / number of women taking folic acid) × 100;
- Folic acid awareness rate (%) = (number of women aware of folic acid benefits / number of women surveyed) × 100;
- NTD incidence rate (per 10 000 perinatal births) = (number of NTD cases / total number of perinatal births) × 10 000.

### Statistical analysis

We used the Cochran–Armitage trend test (linear trend χ^2^) to assess significant trends in the PMC rate, folic acid indicators, and NTDs incidence over the study period. We performed simple linear regression with the PMC rate as the independent variable and NTDs incidence as the dependent variable. We conducted multiple linear regression with the folic acid supplementation rate, compliance rate, and awareness rate as independent variables and NTDs incidence as the dependent variable, while we assessed multicollinearity using variance inflation factors (VIF). We calculated the linear regression coefficient (*β*) along with the corresponding 95% confidence intervals (CIs). We considered a two-sided *P*-value <0.05 to be statistically significant. We compiled data using Microsoft Excel, version 2016 and analysed it using SPSS, version 26.0 (IBM Corp., Armonk, NY, USA).

## RESULTS

### Trends in PMC rate

There were 10 530 233 marriage registrations in Guangxi between 2009 and 2023, of with 9 185 600 (87.23%) couples undergoing PMC. The PMC rate demonstrated a highly significant increasing trend over time (χ^2^ for trend = 2 190 117.322; *P* < 0.001), rising markedly from 14.34% in 2009 to 99.39% in 2023. The most substantial increase occurred between 2009 and 2010 (54.14%). Since 2011, the PMC rate has remained consistently above 90% (**Table 1**).

**Table 1:** PMC rates in Guangxi, 2009–2023

Year	Number of marriage registrations, n	Number of PMC participants	PMC rate, %
2009	1 010 301	144 862	14.34
2010	960 550	657 869	68.48
2011	928 688	874 075	94.12
2012	928 002	899 744	96.95
2013	877 294	855 730	97.54
2014	856 590	836 640	97.67
2015	745 376	731 347	98.12
2016	678 864	667 595	98.34
2017	651 991	644 363	98.83
2018	609 549	603 271	98.97
2019	543 693	539 779	99.30
2020	499 157	496 811	99.53
2021	455 283	453 326	99.56
2022	402 103	399 731	99.41
2023	382 792	380 457	99.39
Total	10 530 233	9 185 600	87.23

PMC – premarital medical check-ups

### Trends in perinatal NTDs incidence

A total of 9 462 842 perinatal infants were monitored during the study period, among which we identified 1082 NTD cases, corresponding to an overall incidence rate of 1.14 per 10 000 individuals. The NTD incidence rate exhibited a significant decreasing trend (χ^2^ for trend = 365.556, *P* < 0.001), declining from 5.44 per 10 000 individuals in 2009 to 0.37 per 10 000 individuals in 2023. We observed the most pronounced decrease between 2010 and 2011 (**Table 2**).

**Table 2:** Perinatal NTDs incidence in Guangxi, 2009–2023

Year	Number of perinatal births	Number of NTD cases	Incidence rate per 10 000 perinatal births
2009	86 425	47	5.44
2010	98 042	52	5.30
2011	819 356	165	2.01
2012	895 701	148	1.65
2013	905 830	149	1.64
2014	860 735	132	1.53
2015	836 928	99	1.18
2016	844 858	76	0.90
2017	825 757	51	0.62
2018	713 656	54	0.76
2019	653 647	39	0.60
2020	577 819	22	0.38
2021	490 426	17	0.35
2022	444 855	16	0.36
2023	408 807	15	0.37
Total	9 462 842	1082	1.14

NTDs – neural tube defects

### Trends in folic acid supplementation, compliance, and awareness rates

Since the implementation of the folic acid prevention project in Guangxi, the folic acid supplementation rate and awareness rate have shown steady annual increases. From 2012 onwards, both the supplementation and awareness rates consistently exceeded 90%, indicating highly effective programme coverage. The folic acid compliance rate also showed a general upward trend (**Table 3**).

**Table 3:** Folic acid supplementation, compliance, and awareness rates in Guangxi, 2009–2023, %

Year	Supplementation rate	Compliance rate	Awareness rate
2009			
2010	57.69	68.75	90.58
2011	86.79	80.14	93.50
2012	93.53	87.34	95.75
2013	92.72	88.41	96.30
2014	96.43	90.88	97.66
2015	97.58	93.20	97.70
2016	98.01	94.67	98.42
2017	100.00	66.76	99.23
2018	100.00	83.46	99.96
2019	100.00	88.46	99.98
2020	100.00	87.58	99.99
2021	100.00	89.26	99.99
2022	100.00	91.10	100.00
2023	100.00	87.92	100.00
χ^2^	143.824	8.678	38.262
*P*-value	<0.001	0.003	<0.001

### Correlation and regression analysis

#### Graphical analysis

**Figure 1:**
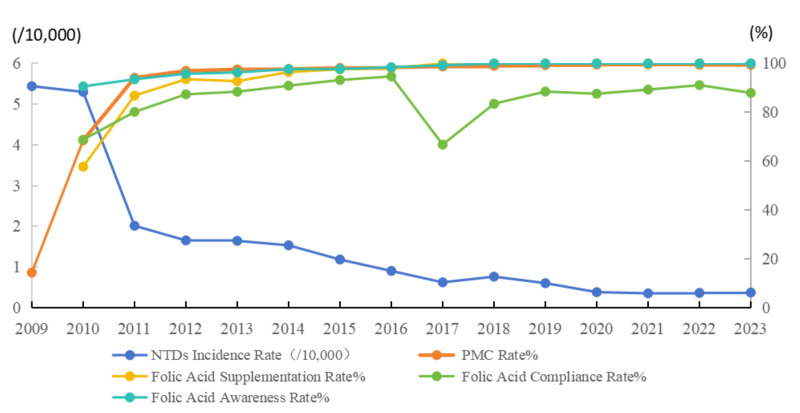
Trends in premarital check-up rate, folic acid-related indicators, and NTDs incidence rate in Guangxi, 2009–2023. NTDs – neural tube defects, PMC – premarital medical check-ups.

The concurrent upward trends in the PMC rate, folic acid supplementation rate, compliance rate, and awareness rate plateaued at high levels. Conversely, the NTD incidence rate shows a clear downward trend, stabilising at a low level over the latter part of the study period (**Figure 1**).

#### Linear regression: PMC rate and NTDs incidence

Simple linear regression with the PMC rate (X_1_) as the independent variable and NTDs incidence (Y) as the dependent variable yielded the equation: Y = 7.278 − 0.063X_1_ (Table S1 in the **Online Supplementary Document**). The model demonstrated good explanatory power, accounting for 74.8% of the variance in NTDs incidence (R^2^ = 0.748). Analysis of variance confirmed the significance of the regression model (F = 38.622, *P* < 0.001). This indicates that the PMC rate significantly influences the NTDs incidence rate; specifically, a 1% increase in the PMC rate is associated with a decrease of 0.063 per 10 000 in the NTDs incidence rate (Table S2 in the **Online Supplementary Document**).

#### Multiple linear regression: folic acid indicators and NTDs incidence

We performed multiple linear regression with folic acid supplementation rate (X_2_), compliance rate (X_3_), and awareness rate (X_4_) as independent variables and NTDs incidence (Y) as the dependent variable. Collinearity diagnostics indicated no significant multicollinearity (all VIF<10). The resulting regression model was Y = 19.564 − 0.090X_2_ (Table S3 in the **Online Supplementary Document**), demonstrating excellent explanatory power and accounting for 97.5% of the variance in NTDs incidence (R^2^ = 0.975). Analysis of variance showed the overall model was significant (F = 131.103, *P* < 0.001; Table S4 in the **Online Supplementary Document**). Among the folic acid indicators, only the supplementation rate had a statistically significant unique contribution to predicting the NTDs incidence rate (*β* = −0.090, *P* < 0.001). A 1% increase in the folic acid supplementation rate was associated with a decrease of 0.090 per 10 000 in the NTDs incidence rate.

## DISCUSSION

We found significant improvements in PMC uptake and folic acid KAP in Guangxi between 2009 and 2023, coinciding with a substantial decline in the incidence of NTDs. The strong negative correlations and predictive relationships found between PMC rate, folic acid supplementation rate, and NTDs incidence highlight the potential synergistic effectiveness of these public health interventions. In contrast, previous studies from regions where comparable premarital health screening systems have not been established or systematically implemented have reported limited declines in NTDs incidence, with rates remaining relatively stable over extended periods [22,23]. For example, population-based longitudinal surveillance data from Scotland [22] showed no significant reduction in overall NTDs incidence between 2000 and 2021 in the absence of mandatory folic acid fortification and without a structured premarital health screening programme, despite the availability of voluntary health education and supplementation recommendations during this period. This finding suggests that, in the absence of institutionalised, population-wide preventive strategies, reliance on individual-level voluntary behaviours alone may be insufficient to achieve substantial reductions in birth defects at the population level. Similarly, in several low-income countries and regions, where standardised premarital health services and comprehensive birth defect prevention programmes are lacking, NTDs have remained at persistently high prevalence levels over time [23]. It should be noted that the decline in NTD incidence observed during the study period is unlikely to be attributable to a single intervention alone. In addition to premarital medical examinations and folic acid supplementation, Guangxi experienced concurrent improvements in socioeconomic development, maternal educational attainment, primary maternal and child health service capacity, and sustained increases in prenatal care coverage over the study period. These factors may have contributed to the observed reduction in NTD incidence at the population level through improvements in periconceptional health behaviours and enhancing risk identification and early intervention [24,25].

The notable success in increasing PMC rates, leading to the nationally recognised ‘Guangxi model’, is attributed to the innovative integrated ‘marriage and childbearing comprehensive service’ platform established in 2010 [26]. This one-stop service model co-locates marriage registration, PMC, preconception care, and antenatal care services, significantly improving accessibility and convenience. Strong governmental commitment, multi-departmental coordination, performance management, sustained funding, and professional training have been key enabling factors.

Regarding folic acid, Guangxi effectively achieved and surpassed the national project's 2011 targets. The high and stable supplementation and awareness rates in recent years reflect successful programme implementation and population coverage. The observed fluctuation in the compliance rate in 2017 (66.76%) warrants attention. This may be related to variations in monitoring intensity, population mobility, or local implementation factors for that specific year, given that the rate recovered subsequently.

Our findings align with previous studies suggesting a beneficial role of PMC in reducing birth defects [27–29]. PMC facilitates early detection and management of health conditions, provides genetic counselling and health education on reproductive topics, and offers a direct channel for distributing folic acid and instructing on its proper use. This creates multiple pathways for NTDs prevention. The protective effect of folic acid supplementation is well-documented globally [30–32], and our results strongly support its role in the observed decline of NTDs in Guangxi.

This study has several limitations. First, although the ecological study design is useful for revealing associations at the population level, the observed associations should be interpreted as reflecting the overall effect of premarital health services rather than the impact of any single component. Moreover, the ecological design inherently limits causal inference and is susceptible to ecological fallacy. Second, as a retrospective analysis relying on routine surveillance data, the findings may be influenced by under-reporting or potential misclassification of NTD cases. Therefore, we cannot fully exclude the potential influence of information bias inherent in long-term surveillance data. In addition, the study population may have included some births to parents who were married before the full implementation of the PMC policy in 2009 and, therefore, were not directly covered by PMC services, particularly during the early phase of policy implementation. Such unavoidable exposure overlap may have influenced the estimated association between PMC coverage and NTDs incidence. Third, owing to limitations in data availability, this study lacked individual-level information on several important potential confounders, including maternal age, detailed nutritional status, and specific environmental exposures. This constrained further adjustment and left room for residual confounding. In addition, we based the analysis on aggregated annual population-level data, and some regression models yielded high correlation coefficients and very small *P*-values. Such statistical patterns are relatively common in time-series or ecological studies and may partly reflect shared temporal trends between the exposure indicators and the incidence of neural tube defects over the study period, as well as the limited number of observation points. Although the data have a clear temporal structure, formal modelling of autocorrelation or lagged effects was not conducted, and residual time-related confounding cannot be fully excluded. Future studies with longer follow-up periods and richer individual-level covariate information should consider segmented time-series regression or multilevel modelling approaches to more robustly evaluate intervention effects. Accordingly, the present findings should be interpreted primarily as statistical associations between population-level trends rather than evidence of direct causal relationships. Despite these limitations, this study provides valuable, long-term, population-based evidence on the association between public health initiatives and NTDs. Future research should prioritise prospective cohort studies with individual-level data collection, incorporating biochemical validation of folic acid status and genetic profiling to better understand the complex interplay between interventions, genetics, and nutrients in preventing NTDs.

## CONCLUSIONS

Over the past 15 years, Guangxi has achieved remarkable progress in promoting premarital medical check-ups and periconceptional folic acid use, concurrent with a significant reduction in the incidence of neural tube defects. The significant negative associations between PMC rate, folic acid supplementation rate, and NTDs incidence suggest that these integrated strategies have contributed substantially to improving birth outcomes. The ‘Guangxi model’ offers a valuable example of a successful, government-led, multi-sectoral approach to preconception care. The continued promotion and strengthening of these interventions are essential. Further optimisation could involve implementing targeted strategies for remote and mobile populations, enhancing follow-up for high-risk individuals, utilising digital health tools for adherence support, and exploring food fortification with folic acid to complement supplementation efforts.

## Additional material


Online Supplementary Document
