# Supplementary material for: Association of premarital medical check-ups and folic acid knowledge, attitudes, and practices with the occurrence of neural tube defects in Guangxi, China (2009–2023)
Source: J Glob Health. 2026 Jun 26;16:04129. doi: 10.7189/jogh.16.04129 (PMC13307441; doi:10.7189/jogh.16.04129)
Supplement: Online Supplementary Document [file jogh-16-04129-s001.pdf]

Supplement to: Liu X, Peng Y, Zhu J. Association of premarital medical check-ups and folic acid knowledge, attitudes, and practices with the occurrence of neural tube defects in Guangxi, China (2009–2003). J Glob Health. 2026;16:04129.

Table S1. Linear Regression Analysis of PMC Rate and NTDs Incidence Rate **Table S2**. ANOVA for Linear Regression Model (PMC Rate vs. NTDs Incidence) **Table S3.** Multiple Linear Regression Analysis of Folic Acid Indicators and NTDs Incidence Rate

Table S4. ANOVA for Multiple Linear Regression Model (Folic Acid Indicators vs. NTDs Incidence)

Text S1. Explanation of authorship change statement

Table S1. Linear Regression Analysis of PMC Rate and NTDs Incidence Rate

**Variable**	**Unstandardized Coefficients (B) (95%CI)**		**Standardized Coefficients**	***t***	***P***
	**B**	**SE**	**β**		
(Constant)	7.278 (5.227, 9.33)	0.950		7.664	<0.001
PMC Rate	-0.063 (-0.085, -0.041)	0.010	-0.865	-6.215	<0.001

PMC – Premarital medical check-ups, NTDs – neural tube defects, CI – confidence interval, SE – standard error

Table S2. ANOVA for Linear Regression Model (PMC Rate vs. NTDs Incidence)

**Source**	**Sum of Squares**	**df**	**Mean Square**	***F***	***P***
Regression	28.424	1	28.424	38.622	<0.001
Residual	9.567	13	0.736		
Total	37.991	14			

PMC – premarital medical check-ups, NTDs – neural tube defects

Table S3. Multiple Linear Regression Analysis of Folic Acid Indicators and NTDs Incidence Rate

**Variable**	**Unstandardized Coefficients (B) (95%CI)**	**SE**	**Standardized Coefficients (β)**	***t***	***P***	**VIF**
(Constant)	19.564 (10.109, 29.018)	4.243		4.611	0.001	
Supplementation Rate	-0.090 (-0.123, -0.057)	0.015	-0.787	-6.032	<0.001	6.868
Compliance Rate	0.004 (-0.016, 0.025)	0.009	0.029	0.476	0.644	1.504
Awareness Rate	-0.104 (-0.225, 0.017)	0.054	-0.232	-1.914	0.085	5.949

NTDs – neural tube defects, CI – confidence interval, SE – standard error

Table S4. ANOVA for Multiple Linear Regression Model (Folic Acid Indicators vs. NTDs Incidence)

**Source**	**Sum of Squares**	**df**	**Mean Square**	***F***	***P***
Regression	21.152	3	7.051	131.103	<0.001
Residual	0.538	10	0.054		
Total	21.689	13			

NTDs – neural tube defects

STROBE Statement—Checklist of items that should be included in reports of ***cross-sectional studies***

	Item No	Recommendation	Page No
**Title and abstract**	1	(*a*) Indicate the study's design with a commonly used term in the title or the abstract	1
		(*b*) Provide in the abstract an informative and balanced summary of what was done and what was found	2-3
**Introduction**			
Background/rationale	2	Explain the scientific background and rationale for the investigation being reported	3-4
Objectives	3	State specific objectives, including any prespecified hypotheses	5
**Methods**			
Study design	4	Present key elements of study design early in the paper	5-9
Setting	5	Describe the setting, locations, and relevant dates, including periods of recruitment, exposure, follow-up, and data collection	5-6
Participants	6	(*a*) Give the eligibility criteria, and the sources and methods of selection of participants	5-6
Variables	7	Clearly define all outcomes, exposures, predictors, potential confounders, and effect modifiers. Give diagnostic criteria, if applicable	8
Data sources/ measurement	8*	For each variable of interest, give sources of data and details of methods of assessment (measurement). Describe comparability of assessment methods if there is more than one group	5-8
Bias	9	Describe any efforts to address potential sources of bias	5-8
Study size	10	Explain how the study size was arrived at	No
Quantitative variables	11	Explain how quantitative variables were handled in the analyses. If applicable, describe which groupings were chosen and why	8-9
Statistical methods	12	(*a*) Describe all statistical methods, including those used to control for confounding	8-9
		(*b*) Describe any methods used to examine subgroups and interactions	No
		(*c*) Explain how missing data were addressed	No
		(*d*) If applicable, describe analytical methods taking account of sampling strategy	No
		(*e*) Describe any sensitivity analyses	No
**Results**			
Participants	13*	(a) Report numbers of individuals at each stage of study—eg numbers potentially eligible, examined for eligibility, confirmed eligible, included in the study, completing follow-up, and analysed	9-10
		(b) Give reasons for non-participation at each stage	No
		(c) Consider use of a flow diagram	No
Descriptive data	14*	(a) Give characteristics of study participants (eg demographic, clinical, social) and information on exposures and potential confounders	9-10
		(b) Indicate number of participants with missing data for each variable of interest	No
Outcome data	15*	Report numbers of outcome events or summary measures	9-10
Main results	16	(*a*) Give unadjusted estimates and, if applicable, confounder-adjusted estimates and their precision (eg, 95% confidence interval). Make clear which confounders were adjusted for and why they were included	10-11

		(*b*) Report category boundaries when continuous variables were categorized	No
		(*c*) If relevant, consider translating estimates of relative risk into absolute risk for a meaningful time period	No
Other analyses	17	Report other analyses done—eg analyses of subgroups and interactions, and sensitivity analyses	No
**Discussion**			
Key results	18	Summarise key results with reference to study objectives	11-13
Limitations	19	Discuss limitations of the study, taking into account sources of potential bias or imprecision. Discuss both direction and magnitude of any potential bias	13-14
Interpretation	20	Give a cautious overall interpretation of results considering objectives, limitations, multiplicity of analyses, results from similar studies, and other relevant evidence	14-15
Generalisability	21	Discuss the generalisability (external validity) of the study results	No
**Other information**			
Funding	22	Give the source of funding and the role of the funders for the present study and, if applicable, for the original study on which the present article is based	15-16

*Give information separately for exposed and unexposed groups.

Note: An Explanation and Elaboration article discusses each checklist item and gives methodological background and published examples of transparent reporting. The STROBE checklist is best used in conjunction with this article (freely available on the Web sites of PLoS Medicine at http://www.plosmedicine.org/, Annals of Internal Medicine at http://www.annals.org/, and Epidemiology at http://www.epidem.com/). Information on the STROBE Initiative is available at www.strobe-statement.org.

Text S1. Explanation of authorship change statement

The authors have requested changes to the order of the authorship byline after submission. Specifically, the initial order was as follows: Xianghong Liu, Jing Zhu.

After the revision process, the authors jointly agreed to revise the authorship byline as follows: Xianghong Liu, Yang Peng, Jing Zhu. Here, Yang Peng was added as the second author.

The authors note that this change emerged after the second round of revisions, when they added YP as the second author, as he contributed to the study design, figure preparation, manuscript writing and proofing. The authors have provided a signed authorship form, a prior version of the manuscript, and proof of correspondence between co-authors as evidence behind this change, and confirm that the requested changes accurately reflect each author's contributions in accordance with the Journal's authorship policies.
